# Should first-line empiric treatment strategies cover coagulase-negative staphylococcal infections in severely malnourished or HIV-infected children in Kenya?

**DOI:** 10.1371/journal.pone.0182354

**Published:** 2017-08-07

**Authors:** Christina W. Obiero, Anna C. Seale, Kelsey Jones, Moses Ngari, Charlotte L. Bendon, Susan Morpeth, Shebe Mohammed, Neema Mturi, Greg Fegan, James A. Berkley

**Affiliations:** 1 Clinical Research Department, KEMRI-Wellcome Trust Research Programme, Kilifi, Kenya; 2 Nuffield Department of Medicine, University of Oxford, Oxford, United Kingdom; 3 Department of Medicine, Imperial College London, London, United Kingdom; 4 Nuffield Department of Obstetrics and Gynaecology, University of Oxford, Oxford, United Kingdom; 5 London School of Hygiene and Tropical Medicine, London, United Kingdom; Universitatsklinikum Hamburg-Eppendorf, GERMANY

## Abstract

**Background:**

Bloodstream infection is a common cause of morbidity in children aged <5 years in developing countries. In studies reporting bacteremia in Africa, coagulase-negative Staphylococci (CoNS) are commonly isolated. However, it is currently unclear whether children who are highly susceptible to infection because of severe acute malnutrition (SAM) or HIV should be treated with antimicrobials specifically to cover CoNS. We aimed to determine the clinical significance of CoNS amongst children admitted to a rural hospital in Kenya in relation to nutritional and HIV status.

**Methods:**

Systematically collected clinical and microbiological surveillance data from children aged 6–59 months admitted to Kilifi County Hospital (2007–2013) were analysed. Multivariable regression was used to test associations between CoNS isolation from blood cultures and SAM (MUAC <11.5cm or nutritional oedema (kwashiorkor)), and HIV serostatus; and among children with SAM or HIV, associations between CoNS isolation and mortality, duration of hospitalization and clinical features.

**Results:**

CoNS were isolated from blood culture in 906/13,315 (6.8%) children, of whom 135/906 (14.9%) had SAM and 54/906 (6.0%) were HIV antibody positive. CoNS isolation was not associated with SAM (MUAC<11.5cm (aOR 1.11, 95% CI 0.88–1.40) or kwashiorkor (aOR 0.84, 95% CI 0.48–1.49)), or a positive HIV antibody test (aOR 1.25, 95% CI 0.92–1.71). Among children with SAM or a positive HIV antibody test, CoNS isolation was not associated with mortality or prolonged hospitalization.

**Conclusion:**

In a large, systematic study, there was no evidence that antimicrobial therapy should specifically target CoNS amongst children with SAM or HIV-infection or exposure.

## Introduction

Infections are amongst the leading causes of morbidity and mortality in children under the age of five years in developing countries despite recent advances in preventive and therapeutic interventions such as vaccines and antimicrobial drugs.[1] Several studies have been conducted in developing countries aimed at determining the causes of bacteraemia in children aged <5 years.[2–6] Findings from these studies have guided vaccine introduction and management of paediatric bacteremia in these settings. However, there still remains a paucity of comprehensive data of potential pathogens causing invasive disease in children in many areas since most health care facilities lack adequate microbiological diagnostic capacity.[3]

Studies of the causes of bacteraemia in hospitalised young children in developing countries have reported that cogulase-negative Staphylococci (CoNS) are frequent blood culture isolates.[2–13] CoNS are variably reported as ‘culture contaminants’ [2, 5–7, 9, 10] or true pathogens.[7, 8, 11–13] However, CoNS are common skin and mucous membranes commensal flora,[14] with relatively low virulence, but possess factors that aid in adherence, biofilm formation, tissue colonization and immune evasion.[15, 16] CoNS are recognized as a cause of clinically significant infections, mostly in preterm infants[17], in critically ill patients with indwelling medical devices[15, 17–19] and in immunocompromised states.[7, 8] It is unknown if CoNS may cause clinically significant infection in children in sub-Saharan Africa with severe acute malnutrition (SAM) and human immunodeficiency virus (HIV) infection who are at an increased risk of serious invasive infection and mortality.[7, 11, 12, 20–24]

One published report from Jamaica has suggested that CoNS are important pathogens amongst children with SAM, and are associated with respiratory signs;[8] and this is referenced in national guidelines for the management of SAM, designed for health facilities that do not have access to microbiological diagnosis.[25] WHO recommends penicillin or ampicillin and gentamicin as first-line antibiotics for complicated severe malnutrition and HIV infected children suspected to have invasive disease. WHO also recommend that teatment regimens need to be adapted to local resistance patterns.[26] A separate study done in Jamaica reported that 6/15 (40%) of blood cuture isolates from 150 severely malnourished children were CoNS, with greater sensitivity to amoxicillin-clavulanic acid than to crystalline penicillin, suggesting that current empiric antimicrobial therapy could be improved.[27] If CoNS are common pathogens in SAM or HIV, then specific treatment with agents such as vacomycin, daptomycin or teicoplanin may be warranted since resistance to beta lactam antibiotics has been demonstrated.[28] Currently, limited data and inconsistent reporting mean that the clinical significance of CoNS is unclear.

To resolve the question of whether CoNS is clinically significant amongst hospitalized children with SAM and/or HIV infection in Africa, we undertook a retrospective analysis of a large systematically collected clinical and microbiological surveillance dataset from hospitalized children in rural Kenya. We aimed to describe the association of CoNS isolation with SAM and HIV status, and association in these groups with clinical signs, mortality, and duration of hospitalization.

## Methods

### Location and participants

The study was conducted at Kilifi County Hospital (KCH), located at the Kenyan coast and serving a mainly rural population of low social economic status. Children requiring hospitalization are admitted to either the general pediatric ward or high dependency unit (HDU) which provides close patient monitoring, but does not have capacity for central venous catheters, arterial lines, parenteral nutrition or invasive ventilation.

All pediatric admissions were systematically assessed and investigated with blood cultures prior to initiation of antibiotics (normally benzylpenicillin or ampicillin plus gentamicin) as previously described.[2] Children presenting with minor trauma or those scheduled for elective surgery did not have blood cultures done and were therefore not included in this analysis. HIV testing was offered to all admissions by trained counsellors, as per Kenya national guidelines.[29] HIV serologic testing was performed using a standard algorithm after consent was obtained from parents or guardians. A positive serologic test [KHB Colloidal Gold (KHB Shanghai Keshaun Bio-engineering Co, Shanghai, China)] was confirmed by a second serologic test [First Response™ 1–2.0 (PMC Medical Pty. Ltd, Daman, India)]. Children with HIV infection or exposure and their families were counselled and referred to the hospital’s HIV clinic for further investigation, management and follow-up. Confirmatory HIV test results by PCR were not consistently available, and we therefore used HIV serologic results for the analysis. Medical care is provided according to World Health Organization (WHO) guidelines for fluids, antimicrobials, nutritional therapy for SAM and other components of supportive care.[26] Clinical and laboratory data were entered routinely onto an electronic data entry system.

All children aged between 6 and 59 months admitted to KCH between January 2007 and December 2013 with blood culture results available were eligible for inclusion into this analysis.

### Clinical definitions

All children underwent anthropometry at admission. SAM was classified according to WHO by the presence of kwashiorkor (nutritional edema), or by mid-upper arm circumference (MUAC) <11.5cm.[26] Features of respiratory distress included nasal flaring, chest wall indrawing, difficulty in breathing, deep respirations, hypoxia or tachypnea. Hypoxia was defined as an oxygen saturation ≤90% in room air using pulse oximetry. Tachypnea was defined as a respiratory rate ≥50 breaths per minute in children aged <12 months, and ≥40 breaths per minute in children aged 12–59 months.[26] Impaired consciousness was defined as Blantyre Coma Scale <4 in children up to 9 months and <5 in children aged ≥9 months.[30]

### Microbiologic methods

Blood samples for culture were collected on admission after children’s skin was cleaned with 70 percent ethanol and allowed to dry as previously described.[2] Regular phlebotomy training was done to reduce blood culture contamination rates. Blood samples were inoculated into commercial vials containing enriched soybean-casein digest broth with CO_2_ (BACTEC Peds Plus/F medium, Becton Dickinson), and incubated in an automated blood culture system (BACTEC 9050, Becton Dickinson). Manufacturer’s recommended blood volume for culture is between 0.5 and 5.0 mL, with optimum results obtained for volumes between 1.0 and 3.0 mL. Blood culture vials were weighed before and after sample inoculation to determine the volume of blood cultured. Culture vials were incubated for 5 days, or until the system indicated the culture was positive. Aliquots of culture sample from positive vials were Gram-stained and sub-cultured, and isolates identified by standard microbiological methods.[2] CoNS, *Bacillus* species, coryneforms, *Micrococcus* species, viridans group streptococci and Candida species were presumed to be contaminants and no targeted therapy for these was given; empiric antibiotic therapy was continued based on clinical resolution of illness/recommended WHO guidelines for use antimicrobials.[26] CoNS species were not determined and CoNS isolates were not stored or tested for antibiotic susceptibilities. All laboratory procedures were internally controlled and KEMRI-Wellcome Trust Research Laboratories were externally monitored for quality assurance by the United Kingdom External Quality Assessment Service and Good Clinical Laboratory Practice (GCLP) accredited by Qualogy, UK.

### Statistical analysis

Statistical analysis was performed using STATA version 13 (Stata Corp., College Station, TX, USA). Data were extracted from the database, checked for any biological implausible measurements that may have resulted from transcription errors, and resolved prior to analysis. Blood culture results were indicated as no growth, CoNS, other isolates presumed non-pathogenic as listed above, or bacterial species that are usually regarded as pathogens (referred to from here as ‘known pathogens’). CoNS were analysed separately from the group of presumed contaminants described above. Children with mixed CoNS and presumed contaminants on blood culture were excluded from this analysis.

Data obtained from initial admission to hospital was included in this analysis; hospital readmission data was not included in this analysis.

Children were grouped by sex, nutritional status (MUAC (≥13.5 cm, 12.5 to 13.4 cm, 11.5 to 12.4 cm, <11.5 cm) or the presence of kwashiorkor) and HIV serologic test result (negative, positive or not performed).

Continuous variables were described using median and interquartile range (IQR) and compared using a Wilcoxon rank sum test. Categorical variables were described using proportions and compared using Chi-squared or Fisher's exact test as appropriate.

Multivariable logistic regression analysis was used to identify putative risk factors of CoNS, and the association of CoNS with clinical signs and symptoms, and mortality. Multivariable linear regression was used to assess the association of CoNS with duration of hospitalization (days). Transformation of age (natural log) and blood volume (square root) was done for the linear regression models.

Regression models were set up using backward selection; predictors with p<0.05, and predictors known a *priori* to act as potential confounders regardless of the p value were included in the final model. For both logistic and linear regression models, adjustment for *a priori* confounders: age, sex and volume of blood culture was done. Inadequate blood samples for culture may increase the likelihood of isolation of CoNS and presumed contaminants, especially in younger children and severely malnourished children with difficult venipuncture. SAM, HIV and malaria are associated with mortaliy in hospitalized children hence these variables were adjusted for in the regression models. Clinical features found to have a p value of ≤0.1 on univariable regression were entered into multivariable regression models to determine the association of CoNS with clinical presentation at admission.

The primary analysis compared children with CoNS isolated to all other children, since the presence of CoNS may have masked growth of known pathogens. As secondary analyses, we also compared children with CoNS isolated to those with no growth on blood cultures, and blood culture isolation of CoNS versus known pathogens. Comparison of children with CoNS to children with presumed contaminants was also done. Sub-group analyses of the relationship between CoNS isolation and HIV status above and below 18 months of age were done as a positive antibody test above 18 months confirms HIV infection, whilst under 18 months, infection and maternal exposure may not be distinguished.

### Ethical considerations

This study was approved by the Kenya Medical Research Institute’s Ethical Review Committee, Nairobi (SSC/ERC 2906). This analysis was a retrospective review of admissions to hospital based on routine clinical data and laboratory findings.

## Results

### Participants and blood culture isolates

Overall, 13,315 children were included in the study (S1 Fig) of whom 1,710 (12.8%) had SAM, and 643 (4.8%) had a positive HIV antibody test (Table 1). CoNS were isolated from blood culture in 906 (6.8%) children, known pathogens were isolated from 564 (4.2%) and 11,044 (82.9%) children had no growth on blood cultures. Presumed contaminants were isolated from blood culture in 801(6.0%) children. 1,714/2,301 (74.5%) of all blood culture isolates were either CoNS or presumed contaminants. 23 children had more than one organism isolated from blood culture (Table 2). None of culture results had CoNS/presumed contaminants isolated together with known pathogens. CoNS isolation declined during the period studied (Table 3).

**Table 1:** Characteristics of study participants.

Characteristic	All children (n = 13,315)^a^	No SAM and HIV (n = 10,171)	SAM only (n = 1,382)	HIV only (n = 315)	SAM and HIV (n = 328)	P value^b^
Age, months^c^	20.0 (11.8–33.5)	20.4 (12.2–34.2)	14.7 (9.6–23.7)	25.8 (15.0–39.6)	19.0(12.4–27.9)	<0.001
Female^d^	5,804 (43.6)	4,343 (42.7)	671 (48.6)	139 (44.1)	156 (47.6)	<0.001
Weight, kg^c^	8.8 (7.2–11.0)	9.2 (7.7–11.3)	6.0 (5.2–7.2)	8.8 (7.1–10.5)	6.0 (5.1–6.9)^b^	<0.001
Height, cm^c^	77.5 (71.0–86.7)	78.5 (72.0–87.7)	70.0 (65.0–76.0)	79.5 (72.4–87.0)	72.0 (66.5–78.0)^b^	<0.001
MUAC, cm^c^	13.5 (12.5–14.6)	14.0 (13.0–15.0)	11.0 (10.0–11.2)	13.0 (12.0–14.0)	10.0 (9.3–11.0)^b^	<0.001
SAM						
MUAC <11.5 cm^d^	1,455 (10.9)	0 (0)	1,136 (82.2)	0 (0)	319 (97.3)	<0.001
Kwashiorkor^d^	255 (1.9)	0 (0)	246 (17.8)	0 (0)	9 (2.7)^b^	
HIV antibody status						
Negative^d^	11,700 (87.9)	10,171 (100)	1,277 (92.4)	0 (0)	0 (0)	<0.001
Positive^d^	643 (4.8)	0 (0)	0 (0)	315 (100)	328 (100)	
Not tested^d^	972 (7.3)	0 (0)	105 (7.6)	0 (0)	0 (0)	
Blood culture results						
No growth^d^	11,044 (82.9)	8,546 (84.0)	1,114 (80.6)	225 (71.4)	220 (67.1)	<0.001
CoNS^d^	906 (6.8)	671 (6.6)	109 (7.9)	28 (8.9)	26 (7.9)	
Known pathogens^d^	564 (4.2)	321 (3.2)	79 (5.7)	45 (14.3)	68 (20.7)	
Presumed contaminants^d^^,^^e^	801 (6.0)	633 (6.2)	80 (5.8)^b^	17 (5.4)	14 (4.3)	
Malaria						
Negative^d^	11,117 (83.5)	8,350 (82.1)	1,289 (93.3)	287 (91.1)	317 (96.7)	<0.001
Positive^d^	2,198 (16.5)	1,821 (17.9)	93 (6.7)	28 (8.9)	11 (3.3)	
Died in hospital^d^	589 (4.4)	229 (2.3)	171 (12.4))	20 (6.4)	75 (22.9)	<0.001
Hospitalization, days^c^	3.0 (2.0–7.0)	3.0 (2.0–5.0)	11.0 (7.0–19.0)	7.0 (4.0–11.0)	18.5 (11.5–28.0)	<0.001

Abbreviations: CoNS, coagulase-negative staphylococci; IQR, interquartile range; MUAC, mid-upper arm circumference; HIV, human immunodeficiency syndrome; SAM, severe acute malnutrition.

^a^ Includes 1,119 children of whom i) 802 did not have SAM but missed HIV status, ii) 252 HIV negative but missed nutrition status, and iii) 65 with both nutrition and HIV status missing.

^b^ Comparison of children without SAM and HIV, children with SAM only, children with HIV only, and children with both SAM and HIV.

^c^ Median (IQR)

^d^ Number (percentage)

^e^ Includes *Bacillus* species, coryneforms, *Micrococcus* species, viridans group streptococci, and Candida species.

**Table 2:** Blood culture isolates from 13,315 children admitted to Kilifi County Hospital between Jan. 2007-Dec. 2013.

	All children (13,315)	SAM only (1,382)	HIV positive only (315)	SAM and HIV positive (328)
Isolates	n = 2,301 (%)^a^	n = 246 (%)^a^	n = 87 (%)^a^	n = 117 (%)^a^
**Gram positive**	**339 (14.7)**	**35 (14.2)**	**32 (36.8)**	**36 (30.8)**
*Streptococcus pneumoniae*	228 (9.9)	25 (10.2)	29 (33.3)	32 (27.4)
*Staphylococcus aureus*	75 (3.3)	6 (2.4)	2 (2.3)	1 (0.9)
Group A Streptococcus	17 (0.7)	0 (0)	1 (1.1)	2 (1.7)
Group D Streptococcus	9 (0.4)	2 (0.8)	0 (0)	0 (0)
Others^b^	10 (0.4)	2 (0.8)	0 (0)	1 (0.9)
**Gram negative**	**248 (10.8)**	**38 (15.4)**	**11 (12.6)**	**40 (34.2)**
*Escherichia coli*	54 (2.3)	13 (5.3)	3 (3.4)	7 (6.0)
Non-typhi Salmonella	53 (2.3)	8 (3.3)	4 (4.6)	14 (12.0)
Acinetobacter sp	38 (1.7)	1 (0.4)	0 (0)	1 (0.9)
*Haemophilus influenzae*	32 (1.4)	3 (1.2)	3 (3.4)	9 (7.7)
*Pseudomonas aeruginosa*	13 (0.6)	4 (1.6)	0	7 (6.0)
*Klebsiella pneumoniae*	10 (0.4)	3 (1.2)	1 (1.1)	1 (0.9)
*Salmonella typhi*	9 (0.4)	0 (0)	0 (0)	0 (0)
*Campylobacter jejuni*	6 (0.3)	3 (1.2)	0 (0)	0 (0)
Others^c^	33 (1.4)	3 (1.2)	0 (0)	1 (0.9)
**CoNS**	**906 (39.4)**	**103 (41.9)**	**27 (31.0)**	**26 (22.2)**
**Presumed contaminants**	**808 (35.1)**	**70 (28.5)**	**17 (19.5)**	**15 (12.8)**
Bacillus sp	624 (27.1)	50 (20.3)	13 (14.9)	9 (7.7)
Coryneforms	93 (4.0)	12 (4.9)	2 (2.3)	3 (2.6)
Micrococcus sp	47 (2.0)	3 (1.2)	0 (0)	0 (0)
*Streptococcus viridans*	39 (1.7)	5 (2.0)	2 (2.3)	3 (2.6)
Candida sp	5 (0.2)	0 (0)	0 (0)	0 (0)

Abbreviations: SAM, severe acute malnutrition; HIV, human immunodeficiency virus; CoNS, coagulase-negative staphylococci.

^a^ Number of isolates (percent): Percentage refers to proportion of isolates in each column (may not sum to 100 due to rounding off).

^b^ Species included streptococci groups C (1), F (4) and G (1), and Streptococcus sp (4).

^c^ Species included Enterobacter sp (5), unidentified Gram negative rods (4), *Pseudomonas stutzeri* (3), *Enterobacter cloacae* (2), *Moraxella catarrhalis* (2), Neisseria sp (2), Pantoea sp (2), *Shigella flexneri* (2), *Acinetobacter lwoffii* (1), Aeromonas sp (1), Bacteroides sp (1), *Enterobacter aerogenes* (1), *Pseudomonas cepacia* (1), *Pseudomonas maltophilia* (1), *Pseudomonas oryzihabitans* (1), *Pseudomonas vescularis* (1), Pseudomonas sp (1), Pasteurella sp (1), and *Shigella sonnei* (1).

**Table 3:** Multivariable analysis of associations with CoNS amongst all admissions.

Characteristics	CoNS (n = 906)	Others (n = 12,409)	No growth (n = 11,044)	Pathogens (n = 564)	CoNS vs Others	CoNS vs No growth	CoNS vs Pathogens
					aOR (95% CI)^a^	aOR (95% CI)^a^	aOR (95% CI)^a^
Age, months	17.7 (10.7–29.9)	20.1 (11.9–33.7)	20.2 (12.0–33.9)	17.8 (10.6–31.0)	0.99 (0.98–0.99)	0.99 (0.98–0.99)	1.00 (0.99–1.00)
Female	368 (40.6)	5, 436 (43.8)	4,840 (43.8)	235 (41.7)	0.88 (0.77–1.01)	0.88 (0.76–1.01)	1.05 (0.83–1.32)
Blood volume, ml	0.8 (0.4–1.3)	0.9 (0.5–1.6)	1.0 (0.5–1.7)	1.0 (0.5–1.7)	0.99 (0.89–1.10)	1.00 (0.90–1.11)	0.76 (0.64–0.90)
Nutritional Status							
MUAC, cm							
≥13.5	471 (52.0)	6,595 (53.2)	5,950 (53.9)	212 (37.6)	Ref	Ref	Ref
12.5–13.4	175 (19.3)	2,480 (20.0)	2,219 (20.1)	96 (17.0)	0.91 (0.76–1.10)	0.91 (0.75–1.10)	0.82 (0.60–1.12)
11.5–12.4	104 (11.5)	1,451 (11.7)	1,261 (11.4)	92 (16.3)	0.90 (0.72–1.13)	0.93 (0.74–1.17)	0.61 (0.43–0.87)
<11.5	122 (13.5)	1,333 (10.7)	1,132 (10.3)	124 (22.0)	1.11 (0.88–1.40)	1.15 (0.91–1.45)	0.65 (0.45–0.92)
Oedema	13 (1.4)	242 (2.0)	202 (1.8)	23 (4.1)	0.84 (0.48–1.49)	0.92 (0.52–1.64)	0.26 (0.13–0.54)
Missing	21 (2.3)	308 (2.4)	280 (2.5)	17 (3.0)	0.82 (0.51–1.31)	0.78 (0.49–1.24)	0.60 (0.29–1.21)
HIV antibody status							
Negative	791 (87.3)	10,909 (87.9)	9,795 (88.7)	402 (71.3)	Ref	Ref	Ref
Positive	54 (6.0)	589 (4.8)	445 (4.0)	113 (20.0)	1.25 (0.92–1.71)	1.49 (1.09–2.04)	0.31 (0.21–0.45)
Not tested	61 (6.7)	911 (7.3)	804 (7.3)	49 (8.7)	1.02 (0.77–1.34)	1.06 (0.80–1.40)	0.64 (0.42–0.97)
Malaria							
Negative	780 86.1)	10,337 (83.3)	9,132 82.7)	522 (92.6)	Ref	Ref	Ref
Positive	126 (13.9)	2,072 (16.7)	1,912 (17.3)	42 (7.4)	0.90 (0.74–1.09)	0.86 (0.70–1.06)	1.91 (1.29–2.82)
Year							
2007	253 (27.9)	2,103 (17.0)	1,743 (15.8)	115 (20.4)	Ref	Ref	Ref
2008	211 (23.3)	1,892 (15.2)	1,571 (14.2)	88 (15.6)	0.89 (0.73–1.09)	0.89 (0.73–1.10)	0.93 (0.65–1.32)
2009	166 (18.3)	2,320 (18.7)	2,047 (18.5)	103 (18.3)	0.59 (0.48–0.72)	0.55 (0.44–0.67)	0.73 (0.52–1.04)
2010	92 (10.2)	1,952 (15.7)	1,785 (16.2)	109 (19.3)	0.40 (0.31–0.52)	0.36 (0.28–0.46)	0.43 (0.29–0.63)
2011	83 (9.2)	1,755 (14.1)	1,655 (15.0)	59 (10.5)	0.39 (0.30–0.51)	0.34 (0.26–0.44)	0.79 (0.51–1.22)
2012	54 (6.0)	1,404 (11.3)	1,321 (12.0)	51 (9.0)	0.32 (0.23–0.45)	0.28 (0.20–0.39)	0.63 (0.38–1.03)
2013	47 (5.2)	983 (7.9)	922 (8.4)	39 (6.9)	0.41 (0.28–0.59)	0.35 (0.24–0.51)	0.81 (0.46–1.43)

Abbreviations: CoNS, Coagulase-negative Staphylococci; OR, odds raio; aOR, adjusted odds ratio; CI, confidence interval; MUAC, mid-upper arm circumference; HIV, human immunodeficiency virus.

^a^ Adjusted for age, sex, blood volume, nutrition status, HIV antibody test, malaria, and year of admission.

Median blood volume cultured was 0.8ml (IQR 0.4–1.3ml) amongst children with CoNS, 1.0ml (IQR 0.5–1.7ml) amongst children with no growth on culture, 0.7ml (IQR 0.3–1.1ml) amongst children with presumed non-pathogenic isolates, and 1.0ml (IQR 0.5–1.7ml) amongst children with known pathogens (S1 Table). The median blood volume cultured was similar in children with CoNS to all others amongst children with SAM [0.8ml (IQR 0.4–1.5ml]) vs. 0.9ml (IQR 0.4–1.6ml), p = 0.10], and amongst children with positive HIV antibody test [0.7ml (IQR 0.3–1.3ml) vs. 0.9ml (IQR 0.4–1.4ml), p = 0.51]. Time to culture positivity amongst children with CoNS (16.2 hours, IQR 14.6–18.4) was less than amongst children with presumed contaminants (36.7 hours, IQR 14.5–65.0), p<0.001.

All children had a blood smear for malaria parasites done at admission with 2,198/13,315 (16.5%) being positive. Although the prevalence of malaria amongst children with CoNS [126/906 (13.9%)] differed from children with either no growth [1,912/11,044 (17.3%), p = 0.01] or known pathogens [42/564 (7.5%), p<0.001], it was similar to prevalence of malaria amongst children with presumed contaminants [118/801 (14.7%), p = 0.63].

### Association between CoNS and SAM

The prevalence of SAM was similar between children with CoNS and all other children (14.9% vs. 12.7%, p = 0.16). There was no evidence of an association between either low MUAC or kwashiorkor and CoNS isolation (S2 Table and Table 3) in multivariable analyses, adjusting for age, sex, blood volume sampled, HIV status, malaria, and year of admission.

Low nutritional status had a strong negative association with isolation of CoNS versus known pathogens [MUAC <11.5cm (aOR 0.65, 95% CI 0.45–0.92) and kwashiorkor (aOR 0.26, 95% CI 0.13–0.54)] (Table 3).

### Association between CoNS and HIV

HIV status did not differ between children with CONS [6.0% (n = 25 aged <18 months and n = 29 aged ≥18 months)] and those without CoNS [4.8% (n = 236 aged <18 months and n = 353 aged ≥18 months), p = 0.22]. Thus, HIV antibody positivity was not associated with CoNS compared with all other children (aOR 1.25, 95% CI 0.92–1.71) (Table 3).

However, HIV antibody positivity was more common amongst children with CoNS than in those with negative cultures, (aOR 1.49, 95% CI 1.09–2.04) but much less common in children with CoNS than in those with known pathogens (aOR 0.31, 95% CI 0.21–0.45) (Table 3).

### Association between CoNS and outcomes in children with SAM or HIV

Amongst children with SAM and children with HIV, there was no association between isolation of CoNS and mortality compared to all other children or children with no growth on culture (Table 4). Children with CoNS were much less likely to die than children with known pathogens, amongst children with SAM (aOR 0.26, 95% CI 0.13–0.49) or positive HIV test (aOR 0.33, 95% CI 0.11–0.98) (Table 4). In both SAM and HIV, children with CoNS were admitted for a similar duration to all other children without CoNS (Table 4).

**Table 4:** Outcomes amongst children with CoNS.

	CoNS	Others	No growth	Pathogens	CoNS vs Others		CoNS vs No growth		CoNS vs Pathogens	
Mortality					OR (95% CI)	aOR (95%CI)	OR (95% CI)	aOR (95% CI)	OR (95% CI)	aOR (95% CI)
All admissions^a^	32 (3.5)	557 (4.5)	420(3.8)	98 (17.4)	0.78 (0.54–1.12)	0.76 (0.52–1.10)^b^	0.93 (0.64–1.34)	0.92 (0.63–1.34)^b^	0.17 (0.11–0.26)	0.20 (0.13–0.33)^a^
SAM^a^	19 (14.1)	227 (14.4)	149 (11.2)	55 (37.7)	0.97 (0.59–1.61)	0.95 (0.57–1.60)^c^	1.30 (0.78–2.18)	1.28 (0.76–2.18)^c^	0.27 (0.15–0.49)	0.26 (0.13–0.49)^b^
Positive HIV test^a^	80 (14.8)	87 (14.9)	50 (11.3)	33 (29.1)	1.00 (0.46–2.19)	0.97 (0.42–2.22)^d^	1.36 (0.61–3.05)	1.42 (0.59–3.38)^d^	0.42 (0.18–0.99)	0.33 (0.11–0.98)^c^
**Duration of hospitalization (survivors), days**					**β coefficient (95% CI)**	**Adjusted β coefficient (95% CI)**	**β coefficient (95% CI)**	**Adjusted β coefficient (95% CI)**	**β coefficient (95% CI)**	**Adjusted β coefficient (95% CI)**
All admissions^e^	3.0 (2.0–7.0)	3.0 (2.0–6.0)	3.0 (2.0–6.0)	8.0 (3.0–15.0)	0.37 (-0.16–0.89)	0.24 (-0.24–0.71)^b^	0.55 (0.03–1.07	0.37 (-0.10–0.84)^b^	-4.80 (-5.79- -3.80)	-3.49 (-4.42- -2.57)^b^
SAM^e^	13.0 (8.0–21.0)	12.0 (7.0–21.0)	12.0 (7.0–20.0)	18.0 (12.0–26.0)	1.22 (-0.97–3.42)	1.03 (-1.11–3.16)^c^	1.57 (-0.64–3.78)	1.36 (-0.79–3.50)^c^	-3.63 (-6.83- -0.43)	-2.72 (-6.10–0.67)^c^
Positive HIV test^e^	11.0 (6.0–15.0)	11.0 (5.0–20.0)	10.0 (5.0–21.0)	12.0 (6.5–20.5)	0.01 (-3.78–3.79)	0.17 (-3.11–3.45)^d^	-0.22 (-4.16–3.71)	-0.05 (-3.54–3.44)^d^	-0.54 (-5.02–3.94)	0.09 (-3.93–4.11)^d^

Abbreviations: CoNS, coagulase-negative staphylococci; OR, odds ratio; CI, confidence interval; aOR, adjusted odds ratio; SAM, severe acute malnutrition; HIV, human immunodeficiency virus.

^a^ n/N (%), number with variable of interest/total group size (percentage).

^b^ Adjusted for age, sex, blood volume, HIV Ab status, nutrition status, malaria, and year.

^c^ Adjusted for age, sex, blood volume, HIV Ab status, malaria, and year.

^d^ Adjusted for age, sex, blood volume, nutrition status, malaria, and year.

^e^ Median (IQR).

### Clinical features of CoNS in children with SAM or HIV

Amongst children with SAM, no clinical signs or symptoms at admission were more frequently observed amongst children with CoNS than all other children after adjusting for age, sex, blood volume sampled, HIV status, malaria, and year of admission (Table 5). Delayed capillary refill was less frequent amongst children with CoNS than amongst all other children. Compared to children with known pathogens, children with CoNS less frequently presented with a prolonged capillary refill time (Table 5).

**Table 5:** Clinical features of CoNS among 1,710 children with severe acute malnutrition.

Characteristic	CoNS n = 135	Others n = 1,575	No growth n = 1,334	Pathogens n = 147	CoNS vs Others		CoNS vs No growth		CoNS vs Pathogens	
	n (%)	n (%)	n (%)	n (%)	OR (95% CI)	aOR (95% CI)^a^	OR (95% CI)	aOR (95% CI)^a^	OR (95% CI)	aOR (95% CI)^b^
History of fever	99 (73.3)	1,130 (72.0)	950 (71.4)	117 (80.1)	1.07 (0.72–1.60)	0.98 (0.63–1.53)	1.10 (0.74–1.64)	1.00 (0.65–1.56)	0.68 (0.39–1.19)	0.69 (0.36–1.36)
Temperature <36.5°C >37.5°C	18 (13.3)67 (49.6)	190 (12.1)758 (48.2)	162 (12.2)629 (47.2)	15 (10.2)81 (55.1)	1.19 (0.68–2.08)1.11 (0.76–1.62)	1.37 (0.77–2.44)1.04 (0.69–1.56)	1.20 (0.68–2.12)1.15 (0.79–1.69)	1.35 (0.75–2.43)1.09 (0.72–1.65)	1.22 (0.56–2.69)0.84 (0.51–1.40)	1.78 (0.69–4.59)1.05 (0.58–1.89)
Cough	90 (66.7)	896 (57.1)	741 (55.7)	105 (71.9)	1.50 (1.04–2.18)	1.28 (0.86–1.90)	1.59 (1.09–2.31)	1.34 (0.90–2.00)	0.78 (0.47–1.30)	0.93 (0.50–1.74)
Respiratory distress	82 (60.7)	875 (55.6)	717 (53.8)	110 (74.8)	1.24 (0.86–1.77)	1.14 (0.75–1.71)	1.33 (0.93–1.91)	1.22 (0.81–1.84)	0.52 (0.31–0.86)	0.72 (0.41–1.30)
Vomiting	42 (31.1)	621 (40.0)	530 (39.9)	56 (38.4)	0.69 (0.47–1.01)	0.79 (0.51–1.24)	0.68 (0.47–0.99)	0.78 (0.49–1.23)	0.73 (0.44–1.19)	0.82 (0.46–1.45)
Diarrhea	51 (37.8)	729 (46.4)	614 (46.2)	68 (46.6)	0.70 (0.49–1.01)	0.81 (0.53–1.25)	0.71 (0.59–1.01)	0.82 (0.53–1.28)	0.70 (0.43–1.12)	0.80 (0.46–1.40)
Inability to drink	4 (3.1)	33 (2.1)	26 (2.0)	6 (4.2)	1.46 (0.51–4.20)	1.44 (0.47–4.40)	1.58 (0.54–4.59)	1.38 (0.44–4.34)	0.74 (0.20–2.67)	1.06 (0.24–4.74)
Capillary refill time, ≥3 sec	2 (1.5)	107 (6.8)	79 (6.0)	19 (13.0)	0.21 (0.05–0.85)	0.23 (0.05–0.95)	0.24 (0.06–0.99)	0.25 (0.06–1.07)	0.10 (0.02–0.45)	0.17 (0.04–0.80)
Convulsions	8 (5.9)	58 (3.7)	44 (3.3)	13 (8.9)	1.64 (0.77–3.52)	1.76 (0.75–4.10)	1.84 (0.85–4.00)	2.11 (0.88–5.07)	0.64 (0.26–1.61)	0.77 (0.26–2.23)
Impaired consciousness	37 (27.6)	376 (24.0)	297 (22.4)	59 (40.4)	1.21 (0.81–1.79)	1.35 (0.88–2.09)	1.32 (0.88–1.97)	1.46 (0.94–2.26)	0.56 (0.34–0.93)	0.63 (0.35–1.14)

^a^ Adjusted for age, sex, blood volume, HIV status, malaria, year, and clinical features with p≤0.1 at univariable regression (cough, vomiting, diarrhea and prolonged capillary refill time).

^b^ Adjusted for age, sex, blood volume, HIV status, malaria, year, and clinical features with p≤0.1 (respiratory distress, prolonged capillary refill time and impaired consciousness).

Amongst children with positive HIV antibody test, no clinical features were more frequent amongst children with CoNS isolation compared with all other children and those with no growth on cultures. Compared to children with known pathogens, children with CoNS were less likely to present with convulsions (aOR 0.16, 95% CI 0.03–0.86) or impaired consciousness level (aOR 0.38, 95% CI 0.15–0.97) (Table 6).

**Table 6:** Clinical features of CoNS among 643 children with a positive HIV antibody test.

Characteristic	CoNS n = 54	Others n = 589	No growth n = 445	Pathogens n = 113	CoNS vs Others		CoNS vs No growth		CoNS vs Pathogens	
	n (%)	n (%)	n (%)	n (%)	OR (95% CI)	aOR (95% CI)^a^	OR (95% CI)	aOR (95% CI)^a^	OR (95% CI)	aOR (95% CI)^b^
History of fever	41 (75.9)	479 (81.5)	354 (79.6)	99 (88.4)	0.72 (0.37–1.39)	0.60 (0.30–1.18)	0.81 (0.42–1.58)	0.70 (0.35–1.39)	0.41 (0.18–0.97)	0.43 (0.15–1.25)
Temperature, <36.5°C >37.5°C	8 (14.8)30 (55.6)	62 (10.6)363 (61.8)	51 (11.5)261 (58.9)	9 (8.0)79 (69.9)	1.31 (0.53–3.21)0.84 (0.44–1.58)	1.43 (0.56–3.64)0.76 (0.39–1.46)	1.28 (0.52–3.19)0.94 (0.50–1.79)	1.36 (0.53–3.52)0.86 (0.44–1.69)	1.39 (0.44–4.34)0.59 (0.28–1.26)	1.84 (0.43–7.85)0.91 (0.35–2.34)
Cough	37 (68.5)	398 (67.8)	284 (64.0)	93 (83.0)	1.03 (0.57–1.88)	0.90 (0.48–1.68)	1.23 (0.67–2.25)	1.11 (0.59–2.09)	0.44 (0.21–0.95)	0.41 (0.15–1.12)
Respiratory distress	39 (72.2)	385 (65.4)	272 (61.1)	93 (82.3)	1.38 (0.74–2.56)	1.30 (0.69–2.46)	1.65 (0.88–3.09)	1.62 (0.85–3.09)	0.56 (0.26–1.20)	0.69 (0.26–1.84)
Vomiting	16 (29.6)	215 (36.6)	169 (38.0)	38 (33.9)	0.73 (0.40–1.34)	0.68 (0.36–1.27)	0.69 (0.37–1.27)	0.65 (0.34–1.24)	0.82 (0.41–1.66)	0.83 (0.37–1.89)
Diarrhea	24 (44.4)	237 (40.3)	188 (42.3)	42 (37.5)	1.18 (0.68–2.08)	1.15 (0.62–2.12)	1.09 (0.62–1.93)	1.07 (0.57–2.02)	1.33 (0.69–2.59)	1.52 (0.66–3.46)
Inability to drink	2 (3.9)	7 (1.21)	5 (1.1)	2 (1.82)	3.27 (0.66–16.20)	2.38 (0.45–12.69)	3.48 (0.66–18.41)	2.15 (0.36–12.67)	2.16 (0.30–15.78)	1.69 (0.14–19.65)
Capillary refill time, ≥3 sec	2 (3.7)	36 (6.1)	26 (5.8)	10 (8.9)	0.59 (0.14–2.52)	0.48 (0.11–2.10)	0.62 (0.14–2.69)	0.49 (0.11–2.26)	0.39 (0.08–1.86)	0.27 (0.05–1.59)
Convulsions	2 (3.7)	44 (7.5)	24 (5.4)	16 (14.3)	0.48 (0.11–2.02)	0.46 (0.10–2.08)	0.67 (0.15–2.94)	0.63 (0.13–3.01)	0.23 (0.05–1.04)	0.16 (0.03–0.86)
Impaired consciousness	9 (16.7)	141 (24.0)	100 (22.5)	37 (33.0)	0.63 (0.30–1.33)	0.61 (0.28–1.31)	0.69 (0.33–1.46)	0.65 (0.29–1.45)	0.41 (0.18–0.92)	0.38 (0.15–0.97)

^a^ Adjusted for age, sex, blood volume, nutrition status, malaria, and year of admission.

^b^ Adjusted for age, sex, blood volume, nutrition status, malaria, year, and clinical features with p≤0.1 at univariable regression (fever, cough, convulsions and impaired consciousness).

### Repeated blood cultures

In our study, 26 of the children who had CoNS isolated at admission had repeat blood cultures because of clinical suspicion of sepsis and poor response to treatment during hospitalization: 14/26 grew CoNS while 6/26 grew known pathogens. 6/26 children died (1 with CoNS) and had the following characteristics: i) SAM, positive HIV antibody test and known pathogen, ii) no SAM, positive HIV antibody test and known pathogen, iii) SAM, negative HIV antibody test and CoNS, iv) SAM and other presumed contaminating isolate, v) negative HIV antibody and other presumed contaminating isolate, and vi) negative HIV antibody test and known pathogen on repeat blood culture. As no speciation of culture isolates was done, it was not known whether repeat CoNS isolates were similar to admission isolates.

## Discussion

In rural Kenya, CoNS were commonly isolated from blood culture amongst children admitted to hospital, as has been reported in several studies in developing countries.[9, 13] However, CoNS was no more common among children with SAM and/or a positive HIV antibody test than among other children. CoNS isolation was not associated with inpatient mortality, or prolonged duration of hospitalization amongst those discharged alive. In children with SAM or HIV there was no clinical phenotype associated with CoNS blood culture isolation. Thus, take the approach of a large epidemiological study, our findings suggests that CoNS are not commonly behaving as pathogens in children with HIV or SAM in this setting, and overall do not warrant targeted empiric first-line therapy. However, we cannot exclude that in a very small number of individuals CoNS may have been pathogenic.

Our conclusions differ to a study conducted among 336 severely malnourished children in Jamaica where CoNS [24/69 (35%)] were the most frequent blood culture isolates. CoNS were reported to be more frequent amongst children with the poorest nutrition status (kwashiorkor and marasmic kwashiorkor) compared to children with marasmus only. Children with CoNS were reported to be more likely to have radiographic pneumonic consolidation than children with bacteremia due to other organisms (8 (22%) p<0.02).[8] CoNS isolation was therefore considered to be clinically significant requiring appropriate antimicrobial therapy. However, surprisingly given the other findings, isolation of CoNS was not associated with mortality. The differences from our study may be due to chance given the small sample size in Jamaica, confounding by differential skin contamination or because children were recruited from a different population.

CoNS have also been reported as common blood culture isolates and of clinical significance amongst hospitalized severely malnourished children in Kenya,[11] Ethiopia,[13] and amongst HIV-infected children in South Africa[12] and Zimbabwe.[7] However, not all of these studies examined clinical outcomes.[11, 12] Others have reported CoNS as ‘contaminants’ in immunocompromised children. Of 846 blood cultures sampled from 461 HIV-infected children in Uganda and Zimbabwe, CoNS [12/127 (9.4%)] were the commonest isolates amongst symptomatic children, but regarded as of ‘doubtful pathogenicity’.[9] CoNS was isolated from 19/38 (50%) of blood culture isolates obtained from 140 severely ill Gambian children with SAM, of whom 27 were HIV-infected. This study was too small to determine an association between CoNS and HIV.[10] One study in Zimbabwe classified pathogenicity of CoNS based on time to positivity <3 days, presence of elevated temperature >38°C or leukocytosis >15x10^9^/liter. Amongst 309 hospitalized children, of whom 168 (54%) were HIV infected, CoNS was the commonest blood culture isolate in all children [31/99 (31%)], and also those who were HIV infected [17/67 (25%)]. Overall, 25/31 (81%) CoNS isolates were assumed to be clinically significant, but again were not associated with mortality.[7]

These findings from resource-poor settings similar to ours reflect the challenges of whether or not to institute definitive management based on interpretation of culture results. Determining whether CoNS are clinically significant in an individual child remains challenging, even when culture facilties exist. For this reason, we chose to examine the risks for CoNS and its outcomes in a large population of sick children in order to inform guidelines used in the vast majority of settings where sick children are treated that do not have access to microbiological diagnosis.

Our study primarily compared children with CoNS to all other children because CoNS may have masked the growth of known pathogens. In addition, we compared children with CoNS to those with either known pathogens or no growth on blood culture. Comparison of CoNS to presumed contaminants did not yield more information than the results we have presented as mortality amongst children with SAM (aOR 0.55, 95%CI 0.25–1.17) and HIV-positive children (aOR 0.83, 95%CI 0.15–4.47), and clinical features did not differ between the two groups.

There were limitations to our study. We did not routinely perform repeat blood cultures when CoNS was isolated or undertake speciation. Our study principally examined blood cultures taken at admission to hospital and therefore may not reflect hospital acquired infections or settings where indwelling devices or invasive ventilation are used. Antibiotic treatment initiated at admission may have influenced outcomes amongst hospitalized children with CoNS if they were sensitive to beta lactams. However, this treatment data was not available and we were therefore not able to adjust for this in the multivariable analysis. We lacked confirmatory PCR test results for HIV viral DNA for children aged <18 months. To mitigate this we conducted a separate analysis adjusting for age (below and above 18 months old) (S3 Table and S4 Table), and results were similar to those obtained including all children with a positive HIV antibody test.

In rural Kenya, although CoNS were common blood culture isolates amongst children with SAM or a positive HIV antibody test, but there was no evidence that CoNS were commonly clinically significant in either of these groups, nor to suggest that specific treatment is required as standard practice.

## Supporting information

S1 Fig Flow diagram. Study participants.
(TIF)

S1 Table Blood volume sampled and time to culture positivity. (DOCX)

S2 Table Univariable analysis of associations with CoNS amongst all admissions. (DOCX)

S3 Table Outcomes amongst children with CoNS. The model includes adjustment of age as a categorical variable (age <18 months or ≥18 months).
(DOCX)

S4 Table Clinical features of CoNS amongst 643 children with a positive HIV antibody test. The model includes adjustment of age as a categorical variable (age <18 months or age ≥18 months).
(DOCX)

## References

[1] Mortality GBD, Causes of Death C. Global, regional, and national age-sex specific all-cause and cause-specific mortality for 240 causes of death, 1990–2013: a systematic analysis for the Global Burden of Disease Study 2013. Lancet. 2015;385(9963):117–71. Epub 2014/12/23. doi: 10.1016/S0140-6736(14)61682-2 ; PubMed Central PMCID: PMCPMC4340604. 25530442 10.1016/S0140-6736(14)61682-2 PMC4340604

[2] Berkley JA, Lowe BS, Mwangi I, Williams T, Bauni E, Mwarumba S, et al Bacteremia among children admitted to a rural hospital in Kenya. The New England journal of medicine. 2005;352(1):39–47. Epub 2005/01/07. doi: 10.1056/NEJMoa040275 . 15635111 10.1056/NEJMoa040275

[3] Isendahl J, Manjuba C, Rodrigues A, Xu W, Henriques-Normark B, Giske CG, et al Prevalence of community-acquired bacteraemia in Guinea-Bissau: an observational study. BMC infectious diseases. 2014;14:3859 Epub 2014/12/21. doi: 10.1186/s12879-014-0715-9 ; PubMed Central PMCID: PMCPMC4297428. 25526763 10.1186/s12879-014-0715-9 PMC4297428

[4] Kibuuka A, Byakika-Kibwika P, Achan J, Yeka A, Nalyazi JN, Mpimbaza A, et al Bacteremia Among Febrile Ugandan Children Treated with Antimalarials Despite a Negative Malaria Test. Am J Trop Med Hyg. 2015;93(2):276–80. doi: 10.4269/ajtmh.14-0494 ; PubMed Central PMCID: PMCPMC4530747. 26055736 10.4269/ajtmh.14-0494 PMC4530747

[5] Dramowski A, Cotton MF, Rabie H, Whitelaw A. Trends in paediatric bloodstream infections at a South African referral hospital. BMC pediatrics. 2015;15:33 Epub 2015/04/18. doi: 10.1186/s12887-015-0354-3 ; PubMed Central PMCID: PMCPMC4396163. 25884449 10.1186/s12887-015-0354-3 PMC4396163

[6] Nielsen MV, Sarpong N, Krumkamp R, Dekker D, Loag W, Amemasor S, et al Incidence and characteristics of bacteremia among children in rural Ghana. PLoS One. 2012;7(9):e44063 Epub 2012/09/13. doi: 10.1371/journal.pone.0044063 ; PubMed Central PMCID: PMCPMC3438186. 22970162 10.1371/journal.pone.0044063 PMC3438186

[7] Nathoo KJ, Chigonde S, Nhembe M, Ali MH, Mason PR. Community-acquired bacteremia in human immunodeficiency virus-infected children in Harare, Zimbabwe. The Pediatric infectious disease journal. 1996;15(12):1092–7. Epub 1996/12/01. . 10.1097/00006454-199612000-00007 8970218

[8] Christie CD, Heikens GT, Golden MH. Coagulase-negative staphylococcal bacteremia in severely malnourished Jamaican children. The Pediatric infectious disease journal. 1992;11(12):1030–6. Epub 1992/12/01. . 1461693 10.1097/00006454-199211120-00008

[9] Musiime V, Cook A, Bakeera-Kitaka S, Vhembo T, Lutakome J, Keishanyu R, et al Bacteremia, causative agents and antimicrobial susceptibility among HIV-1-infected children on antiretroviral therapy in Uganda and Zimbabwe. The Pediatric infectious disease journal. 2013;32(8):856–62. Epub 2013/02/15. doi: 10.1097/INF.0b013e31828c3991 . 23407100 10.1097/INF.0b013e31828c3991

[10] Okomo UA, Garba D, Fombah AE, Secka O, Ikumapayi UN, Udo JJ, et al Bacterial Isolates and Antibiotic Sensitivity among Gambian Children with Severe Acute Malnutrition. International journal of pediatrics. 2011;2011:825123 Epub 2011/07/26. doi: 10.1155/2011/825123 ; PubMed Central PMCID: PMC3139120. 21785610 10.1155/2011/825123 PMC3139120

[11] Noorani N, Macharia WM, Oyatsi D, Revathi G. Bacterial isolates in severely malnourished children at Kenyatta National Hospital, Nairobi. East African medical journal. 2005;82(7):343–8. Epub 2005/09/20. . 16167706

[12] Jaspan HB, Huang LC, Cotton MF, Whitelaw A, Myer L. Bacterial disease and antimicrobial susceptibility patterns in HIV-infected, hospitalized children: a retrospective cohort study. PLoS One. 2008;3(9):e3260 Epub 2008/09/25. doi: 10.1371/journal.pone.0003260 ; PubMed Central PMCID: PMC2533121. 18813340 10.1371/journal.pone.0003260 PMC2533121

[13] Abrha A, Abdissa A, Beyene G, Getahun G, Girma T. Bacteraemia among severely malnourished children in jimma university hospital, ethiopia. Ethiop J Health Sci. 2011;21(3):175–82. Epub 2012/03/22. ; PubMed Central PMCID: PMC3275866. 22434997 PMC3275866

[14] Costa SF, Miceli MH, Anaissie EJ. Mucosa or skin as source of coagulase-negative staphylococcal bacteraemia? The Lancet Infectious Diseases. 2004;4(5):278–86. doi: 10.1016/S1473-3099(04)01003-5 15120344 10.1016/S1473-3099(04)01003-5

[15] Giormezis N, Kolonitsiou F, Foka A, Drougka E, Liakopoulos A, Makri A, et al Coagulase-negative staphylococcal bloodstream and prosthetic-device-associated infections: the role of biofilm formation and distribution of adhesin and toxin genes. Journal of medical microbiology. 2014;63(Pt 11):1500–8. Epub 2014/08/02. doi: 10.1099/jmm.0.075259-0 . 25082946 10.1099/jmm.0.075259-0

[16] Otto M. Staphylococcus epidermidis—the 'accidental' pathogen. Nature reviews Microbiology. 2009;7(8):555–67. Epub 2009/07/18. doi: 10.1038/nrmicro2182 ; PubMed Central PMCID: PMC2807625. 19609257 10.1038/nrmicro2182 PMC2807625

[17] Marchant EA, Boyce GK, Sadarangani M, Lavoie PM. Neonatal sepsis due to coagulase-negative staphylococci. Clinical & developmental immunology. 2013;2013:586076 Epub 2013/06/14. doi: 10.1155/2013/586076 ; PubMed Central PMCID: PMC3674645. 23762094 10.1155/2013/586076 PMC3674645

[18] Huebner M, Johannes, Goldmann M, Donald A. Coagulase-negative staphylococci: role as pathogens. Annual review of medicine. 1999;50(1):223–36. 10.1146/annurev.med.50.1.223 10073274

[19] Huang SY, Tang RB, Chen SJ, Chung RL. Coagulase-negative staphylococcal bacteremia in critically ill children: risk factors and antimicrobial susceptibility. Journal of microbiology, immunology, and infection = Wei mian yu gan ran za zhi. 2003;36(1):51–5. Epub 2003/05/14. . 12741734

[20] Meyers TM, Pettifor JM, Gray GE, Crewe-Brown H, Galpin JS. Pediatric admissions with human immunodeficiency virus infection at a regional hospital in Soweto, South Africa. Journal of tropical pediatrics. 2000;46(4):224–30. Epub 2000/09/21. . 10996984 10.1093/tropej/46.4.224

[21] Bachou H, Tylleskar T, Kaddu-Mulindwa DH, Tumwine JK. Bacteraemia among severely malnourished children infected and uninfected with the human immunodeficiency virus-1 in Kampala, Uganda. BMC infectious diseases. 2006;6:160 Epub 2006/11/09. doi: 10.1186/1471-2334-6-160 ; PubMed Central PMCID: PMC1660577. 17090299 10.1186/1471-2334-6-160 PMC1660577

[22] Babirekere-Iriso E, Musoke P, Kekitiinwa A. Bacteraemia in severely malnourished children in an HIV-endemic setting. Annals of tropical paediatrics. 2006;26(4):319–28. Epub 2006/11/30. doi: 10.1179/146532806X152845 . 17132297 10.1179/146532806X152845

[23] Rabasa AI, Shattima D. Urinary tract infection in severely malnourished children at the University of Maiduguri Teaching Hospital. Journal of tropical pediatrics. 2002;48(6):359–61. Epub 2003/01/11. . 12521279 10.1093/tropej/48.6.359

[24] Aiken AM, Mturi N, Njuguna P, Mohammed S, Berkley JA, Mwangi I, et al Risk and causes of paediatric hospital-acquired bacteraemia in Kilifi District Hospital, Kenya: a prospective cohort study. Lancet. 2011;378(9808):2021–7. doi: 10.1016/S0140-6736(11)61622-X ; PubMed Central PMCID: PMCPMC3242162. 22133536 10.1016/S0140-6736(11)61622-X PMC3242162

[25] Ministry of Medical Services. National Guideline for Integrated Management of Acute Malnutrition. Version 1. June 2009. [accessed 23 Aug 2015]. Available from: http://www.cmamforum.org/pool/resources/kenya-moh-imam-guideline-june-2009.pdf.

[26] WHO. Pocket book of hospital care for children: WHO Press; 2013 Available from: http://www.who.int/maternal_child_adolescent/documents/child_hospital_care/en/.

[27] Thame M, Stephen C, Wilks R, Forrester TE. The appropriateness of the current antibiotic empiric therapy based on the bacteria isolated from severely malnourished Jamaican children. The West Indian medical journal. 2001;50(2):140–3. Epub 2001/10/27. . 11677912

[28] Morad Asaad A, Ansar Qureshi M, Mujeeb Hasan S. Clinical significance of coagulase-negative staphylococci isolates from nosocomial bloodstream infections. Infect Dis (Lond). 2016;48(5):356–60. doi: 10.3109/23744235.2015.1122833 . 26666168 10.3109/23744235.2015.1122833

[29] National AIDS and STI Control Programme MoPHaS, Kenya. Guidelines for HIV Testing and Counselling in Kenya. Nairobi, Kenya2010. Available from: http://nascop.or.ke/library/HTC/National%20Guidelines%20for%20HTC%20in%20Kenya%202010.pdf.

[30] Molyneux ME, Taylor TE, Wirima JJ, Borgstein A. Clinical features and prognostic indicators in paediatric cerebral malaria: a study of 131 comatose Malawian children. The Quarterly journal of medicine. 1989;71(265):441–59. Epub 1989/05/01. . 2690177

