# Supplementary figures and images for: Should first-line empiric treatment strategies cover coagulase-negative staphylococcal infections in severely malnourished or HIV-infected children in Kenya?
Source: PLoS One. 2017 Aug 7;12(8):e0182354. doi: 10.1371/journal.pone.0182354 (PMC5546690; doi:10.1371/journal.pone.0182354)

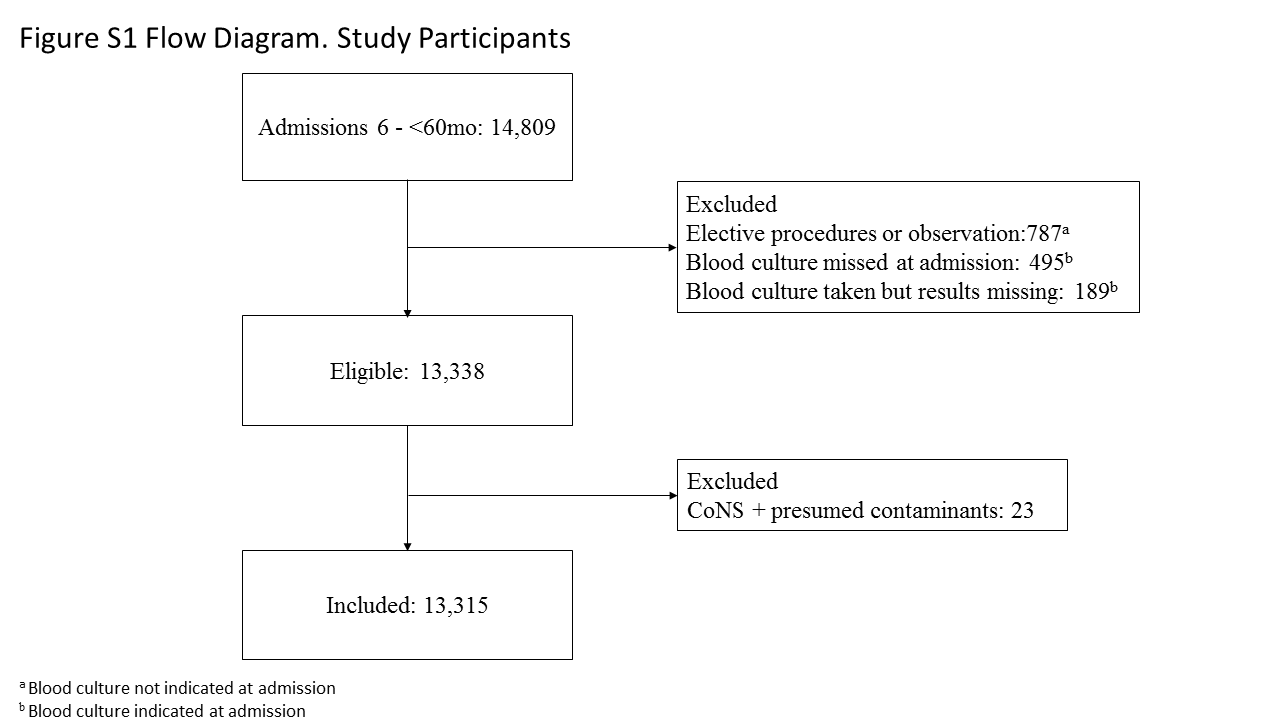

Supplement: S1 Fig — Study participants. (TIF) [file pone.0182354.s001.tif]
