# Supplementary material for: Should first-line empiric treatment strategies cover coagulase-negative staphylococcal infections in severely malnourished or HIV-infected children in Kenya?
Source: PLoS One. 2017 Aug 7;12(8):e0182354. doi: 10.1371/journal.pone.0182354 (PMC5546690; doi:10.1371/journal.pone.0182354)
Supplement: S1 Table — (DOCX) [file pone.0182354.s002.docx]

## Table S1. Blood volume sampled and time to culture positivity

| **Characteristic** | **All children**  **(n=13,315)** | **No SAM and HIV (n=10,171)** | **SAM only (n=1,382)** | **HIV positive only**  **(n=315)** | **SAM HIV positive (n=328)** | **P value^a^** |
| --- | --- | --- | --- | --- | --- | --- |
| Blood volume cultured, ml^b^ | 0.9  (0.5-1.6) | 0.9  (0.5-1.6) | 0.9  (0.4-1.6) | 0.8  (0.4-1.4) | 0.9  (0.4-1.5) | 0.033 |
| Time to positivity, hrs^b^ | 15.8  (11.2-24.0) | 16.0  (11.9-22.3) | 14.2  (11.2-18.4) | 11.6  (8.9-15.9) | 12.8  (10.2-17.8) | 0.004 |
|  |  | **CoNS (n=906)** | **No growth (n=11,044)** | **Known pathogens (n=564)** | **Presumed contaminants (n=801)** |  |
| Blood volume, ml^a^ |  | 0.8  (0.4-1.3) | 1.0  (0.5-1.7) | 1.0  (0.5-1.7) | 0.7  (0.3-1.1) | <0.001 |
| Time to positivity, hrs^a^ |  | 16.2  (14.6-18.4) | - | 10.8  (8.8-15.5) | 36.7  (14.5-65.0) | <0.001 |

Abbreviations: CoNS, coagulase-negative staphylococci; IQR, interquartile range; HIV, human immunodeficiency syndrome; SAM, severe acute malnutrition.

^a^ Comparison across groups

^b^ Median (IQR)
