# Supplementary material for: Should first-line empiric treatment strategies cover coagulase-negative staphylococcal infections in severely malnourished or HIV-infected children in Kenya?
Source: PLoS One. 2017 Aug 7;12(8):e0182354. doi: 10.1371/journal.pone.0182354 (PMC5546690; doi:10.1371/journal.pone.0182354)
Supplement: S2 Table — (DOCX) [file pone.0182354.s003.docx]

## Table S2. Univariable Analysis of Associations with CoNS amongst all Admissions

| **Characteristics** | **CoNS**  **(n=906)** | **Others**  **(n=12,409)** | **No growth**  **(n=11,044)** | **Pathogens**  **(n=564)** | **CoNS vs Others** | **CoNS vs No growth** | **CoNS vs Pathogens** |
| --- | --- | --- | --- | --- | --- | --- | --- |
|  |  |  |  |  | OR (95% CI) | OR (95% CI) | OR (95% CI) |
| Age, months | 17.7 (10.7-29.9) | 20.1 (11.9-33.7) | 20.2 (12.0-33.9) | 17.8 (10.6-31.0) | 0.99 (0.98-0.99) | 0.99 (0.98-0.99) | 0.99 (0.98-1.01) |
| Female | 368 (40.6) | 5, 436 (43.8) | 4,840 (43.8) | 235 (41.7) | 0.88 (0.76-1.01) | 0.88 (0.76-1.01) | 0.96 (0.77-1.19) |
| Blood volume, ml | 0.8 (0.4-1.3) | 0.9 (0.5-1.6) | 1.0 (0.5-1.7) | 1.0 (0.5-1.7) | 0.76 (0.70-0.84) | 0.74 (0.68-0.81) | 0.69 (0.61-0.79) |
| Nutritional Status |  |  |  |  |  |  |  |
| MUAC, cm |  |  |  |  |  |  |  |
| ≥13.5 | 471 (52.0) | 6,595 (53.2) | 5,950 (53.9) | 212 (37.6) | Ref | Ref | Ref |
| 12.5-13.4 | 175 (19.3) | 2,480 (20.0) | 2,219 (20.1) | 96 (17.0) | 0.99 (0.83-1.18) | 1.00 (0.83-1.19) | 0.82 (0.61-1.10) |
| 11.5-12.4 | 104 (11.5) | 1,451 (11.7) | 1,261 (11.4) | 92 (16.3) | 1.00 (0.81-1.25) | 1.04 (0.84-1.30) | 0.51 (0.37-0.70) |
| <11.5 | 122 (13.5) | 1,333 (10.7) | 1,132 (10.3) | 124 (22.0) | 1.28 (1.04-1.58) | 1.36 (1.10-1.68) | 0.44 (0.33-0.60) |
| Oedema | 13 (1.4) | 242 (2.0) | 202 (1.8) | 23 (4.1) | 0.75 (0.43-1.32) | 0.81 (0.46-1.44) | 0.25 (0.13-0.51) |
| Missing | 21 (2.3) | 308 (2.4) | 280 (2.5) | 17 (3.0) | 0.95 (0.61-1.50) | 0.95 (0.60-1.49) | 0.56 (0.29-1.08) |
| HIV antibody status |  |  |  |  |  |  |  |
| Negative | 791 (87.3) | 10,909 (87.9) | 9,795 (88.7) | 402 (71.3) | Ref | Ref | Ref |
| Positive | 54 (6.0) | 589 (4.8) | 445 (4.0) | 113 (20.0) | 1.26 (0.95-1.69) | 1.50 (1.12-2.01) | 0.24 (0.17-0.34) |
| Not tested | 61 (6.7) | 911 (7.3) | 804 (7.3) | 49 (8.7) | 0.92 (0.71-1.21) | 0.94 (0.72-1.23) | 0.63 (0.43-0.94) |
| Malaria |  |  |  |  |  |  |  |
| Negative | 780 (86.1) | 10,337 (83.3) | 9,132 (82.7) | 522 (92.6) | Ref | Ref | Ref |
| Positive | 126 (13.9) | 2,072 (16.7) | 1,912 (17.3) | 42 (7.4) | 0.81 (0.66-0.98) | 0.77 (0.64-0.94) | 2.01 (1.39-2.90) |
| Year |  |  |  |  |  |  |  |
| 2007 | 253 (27.9) | 2,103 (17.0) | 1,743 (15.8) | 115 (20.4) | Ref | Ref | Ref |
| 2008 | 211 (23.3) | 1,892 (15.2) | 1,571 (14.2) | 88 (15.6) | 0.93 (0.76-1.12) | 0.93 (0.76-1.12) | 1.09 (0.78-1.52) |
| 2009 | 166 (18.3) | 2,320 (18.7) | 2,047 (18.5) | 103 (18.3) | 0.59 (0.48-0.73) | 0.56 (0.45-0.69) | 0.73 (0.53-1.02) |
| 2010 | 92 (10.2) | 1,952 (15.7) | 1,785 (16.2) | 109 (19.3) | 0.39 (0.31-0.50) | 0.36 (0.28-0.45) | 0.38 (0.27-0.55) |
| 2011 | 83 (9.2) | 1,755 (14.1) | 1,655 (15.0) | 59 (10.5) | 0.39 (0.30-0.51) | 0.35 (0.27-0.45) | 0.64 (0.43-0.95) |
| 2012 | 54 (6.0) | 1,404 (11.3) | 1,321 (12.0) | 51 (9.0) | 0.32 (0.24-0.43) | 0.28 (0.21-0.38) | 0.48 (0.31-0.75) |
| 2013 | 47 (5.2) | 983 (7.9) | 922 (8.4) | 39 (6.9) | 0.40 (0.29-0.55) | 0.35 (0.25-0.48) | 0.55 (0.34-0.88) |

Abbreviations: CoNS, Coagulase-negative Staphylococci; OR, odds ratio; CI, confidence interval; MUAC, mid-upper arm circumference; HIV, human immunodeficiency virus.
