# Supplementary material for: Should first-line empiric treatment strategies cover coagulase-negative staphylococcal infections in severely malnourished or HIV-infected children in Kenya?
Source: PLoS One. 2017 Aug 7;12(8):e0182354. doi: 10.1371/journal.pone.0182354 (PMC5546690; doi:10.1371/journal.pone.0182354)
Supplement: S3 Table — The model includes adjustment of age as a categorical variable (age <18 months or ≥18 months). (DOCX) [file pone.0182354.s004.docx]

## Table S3. Outcomes amongst children with CoNS

|  | **CoNS** | **Others** | **No growth** | **Pathogens** | **CoNS vs Others** | | **CoNS vs No growth** | | **CoNS vs Pathogens** | |
| --- | --- | --- | --- | --- | --- | --- | --- | --- | --- | --- |
| **Mortality** |  |  |  |  | **OR (95%CI)** | **aOR (95%CI)** | **OR (95% CI)** | **aOR (95% CI)** | **OR (95% CI)** | **aOR (95% CI)** |
| All admissions^a^ | 32 (3.5) | 557 (4.5) | 420 (3.8) | 98 (17.4) | 0.78  (0.54-1.12) | 0.75  (0.52-1.10)^b^ | 0.93  (0.64-1.34) | 0.91  (0.62-1.33)^b^ | **0.17**  **(0.11-0.26)** | **0.20**  **(0.13-0.33)^b^** |
| SAM^a^ | 19 (14.1) | 227 (14.4) | 149 (11.2) | 55 (37.7) | 0.97  (0.59-1.61) | 0.95  (0.57-1.60)^c^ | 1.30  (0.78-2.18) | 1.29  (0.76-2.19)^c^ | **0.27**  **(0.15-0.49)** | **0.25**  **(0.13-0.49)^c^** |
| Positive HIV test^a^ | 8.0 (14.8) | 87 (14.9) | 50 (11.3) | 33 (29.2) | 1.00  (0.46-2.19) | 0.97  (0.42-2.23)^d^ | 1.36  (0.61-3.05) | 1.43  (0.60-3.41)^d^ | **0.42**  **(0.18-0.99)** | 0.35  (0.12-1.01)^d^ |
| **Duration of hospitalization (survivors), days** |  |  |  |  | **β coefficient**  **(95% CI)** | **Adjusted β coefficient**  **(95% CI)** | **β coefficient**  **(95% CI)** | **Adjusted β coefficient**  **(95% CI)** | **β**  **coefficient (95%CI)** | **Adjusted β coefficient**  **(95% CI)** |
| All admissions^e^ | 3.0  (2.0-7.0) | 3.0  (2.0-6.0) | 3.0  (2.0-6.0) | 8.0  (3.0-15.0) | 0.37  (-0.16-0.89) | 0.21  (-0.27-0.68)^b^ | **0.55**  **(0.03-1.07**) | 0.33  (-0.14-0.80)^b^ | **-4.80**  **(-5.79- -3.80)** | **-3.49**  **(-4.42- -2.57)^b^** |
| SAM^e^ | 13.0  (8.0-21.0) | 12.0  (7.0-21.0) | 12.0  (7.0-20.0) | 18.0  (12.0-26.0) | 1.22  (-0.97-3.42) | 0.87  (-1.28-3.01)^c^ | 1.57  (-0.64-3.78) | 1.17  (-0.99-3.33)^c^ | **-.3.63**  **(-6.83- -0.43)** | -2.93  (-6.38-0.51)^c^ |
| Positive HIV test^e^ | 11.0  (6.0-15.0) | 11.0  (5.0-20.0) | 10.0  (5.0-21.0) | 12.0  (6.5-20.5) | 0.01  (-3.78-3.79) | 0.12  (-3.17-3.40)^d^ | -0.22  (-4.16-3.71) | -0.13  (-3.63-3.38)^d^ | -0.54  (-5.02-3.94) | 0.03  (-4.03-4.09)^d^ |

Abbreviations: CoNS, coagulase-negative staphylococci; OR, odds ratio; CI, confidence interval; n/N, number with variable of interest/total group size; SAM, severe acute malnutrition; HIV, human immunodeficiency virus.

^a^ n/N (%), number with variable of interest/total group size (percentage)

^b^ Adjusted for age (<18 months or ≥18 months), sex, blood volume, HIV status, nutrition status, malaria, and year.

^c^ Adjusted for age (<18 months or ≥18 months), sex, blood volume, HIV status, malaria, and year.

^d^ Adjusted for age (<18 months or ≥18 months), sex, blood volume, nutrition status, malaria, and year.

^e^ Median (IQR)
