# Supplementary material for: Should first-line empiric treatment strategies cover coagulase-negative staphylococcal infections in severely malnourished or HIV-infected children in Kenya?
Source: PLoS One. 2017 Aug 7;12(8):e0182354. doi: 10.1371/journal.pone.0182354 (PMC5546690; doi:10.1371/journal.pone.0182354)
Supplement: S4 Table — The model includes adjustment of age as a categorical variable (age <18 months or age ≥18 months). (DOCX) [file pone.0182354.s005.docx]

## Table S4. Clinical features of CoNS among 643 children with a positive HIV antibody test

| **Characteristic** | **CoNS n=54**  **(%)** | **Others n=589**  **(%)** | **No growth n=445**  **(%)** | **Pathogens n=113**  **(%)** | **CoNS vs Others** | | **CoNS vs No growth** | | **CoNS vs Pathogens** | |
| --- | --- | --- | --- | --- | --- | --- | --- | --- | --- | --- |
|  |  |  |  |  | **OR**  **(95% CI)** | **aOR**  **(95% CI)^a^** | **OR**  **(95% CI)** | **aOR**  **(95% CI)^a^** | **OR**  **(95% CI)** | **aOR**  **(95% CI)^b^** |
| History of fever | 41 (75.9) | 479 (81.5) | 354 (79.6) | 99 (88.4) | 0.72  (0.37-1.39) | 0.59  (0.30-1.18) | 0.81  (0.42-1.58) | 0.70  (0.35-1.40) | 0.41  (0.18-0.97) | 0.43  (0.14-1.26) |
| Temperature,  <36.5⁰C  >37.5⁰C | 8 (14.8)  30 (55.6) | 62 (10.6)  363 (61.8) | 51 (11.5)  261 (58.9) | 9 (8.0)  79 (69.9) | 1.31  (0.53-3.21)  0.84  (0.44-1.58) | 1.47  (0.57-3.76)  0.76  (0.39-1.46) | 1.28  (0.52-3.19)  0.94  (0.50-1.79) | 1.39  (0.54-3.60)  0.87  (0.44-1.69) | 1.39  (0.44-4.34)  0.59  (0.28-1.26) | 1.88  (0.44-8.11)  0.89  (0.35-2.29) |
| Cough | 37 (68.5) | 398 (67.8) | 28 (64.0) | 93 (83.0) | 1.03  (0.57-1.88) | 0.90  (0.48-1.69) | 1.23  (0.67-2.25) | 1.11  (0.59-2.10) | 0.44  (0.21-0.95) | 0.41  (0.15-1.12) |
| Respiratory distress | 39 (72.2) | 385 (65.4) | 272 (61.1) | 93 (82.3) | 1.38  (0.74-2.57) | 1.30  (0.69-2.46) | 1.65  (0.88-3.09) | 1.62  (0.85-3.10) | 0.56  (0.26-1.20) | 0.70  (0.27-1.85) |
| Vomiting | 16 (29.6) | 215 (36.6) | 169 (38.0) | 38 (33.9) | 0.73  (0.40-1.34) | 0.67  (0.36-1.27) | 0.69  (0.37-1.27) | 0.65  (0.34-1.24) | 0.82  (0.41-1.66) | 0.82  (0.36-1.87) |
| Diarrhea | 24 (44.4) | 237 (40.3) | 188 (42.3) | 42 (37.5) | 1.18  (0.68-2.08) | 1.15  (0.62-2.12) | 1.09  (0.62-1.93) | 1.07  (0.57-2.01) | 1.33  (0.69-2.59) | 1.54  (0.68-3.51) |
| Inability to drink | 2 (3.9) | 7 (1.21) | 5 (1.1) | 2 (1.82) | 3.27  (0.66-16.20) | 2.39  (0.45-12.72) | 3.48  (0.66-18.41) | 2.17  (0.37-12.80) | 2.16  (0.30-15.78) | 1.64  (0.14-19.56) |
| Capillary refill time, ≥3 sec | 2 (3.7) | 36 (6.1) | 26 (5.8) | 10 (8.9) | 0.59  (0.14-2.52) | 0.48  (0.11-2.10) | 0.62  (0.14-2.69) | 0.48  (0.10-2.22) | 0.39  (0.08-1.86) | 0.29  (0.05-1.65) |
| Convulsions | 2 (3.7) | 44 (7.5) | 24 (5.4) | 16 (14.3) | 0.48  0.11-2.02) | 0.46  (0.10-2.07) | 0.67  (0.15-2.94) | 0.64  (0.13-3.04) | 0.23  (0.05-1.04) | 0.16  (0.03-0.85) |
| Impaired consciousness | 9 (16.7) | 141(24.0) | 100 (22.5) | 37 (33.0) | 0.63  (0.30-1.33) | 0.61  (0.28-1.32) | 0.69  (0.33-1.46) | 0.66  (0.30-1.46) | 0.41  (0.18-0.92) | 0.39  (0.15-0.98) |

^a^ Adjusted for age (<18 months or ≥18 months), sex, blood volume, nutrition status and year of admission.

^b^ Adjusted for age (<18 months or ≥18 months), sex, blood volume, nutrition status, year and clinical features with p<0.1 at univariable regression (fever, cough, convulsions and impaired consciousness).
